# The influence of medical insurance and social security cards on the floating population's settlement intention

**DOI:** 10.1186/s12962-021-00321-4

**Published:** 2021-10-09

**Authors:** Yulin Li, Lingling Huang, Li Xiang, Dongmei Dou

**Affiliations:** 1 School of Nursing and Health, Institute for Chronic Disease Risk Assessment, Henan University, Kaifeng, 475004 China; 2 School of Nursing and Health, Lida University, Shanghai, 201609 China; 3 School of Medicine and Health Management, Tongji Medical College of Huazhong University of Science and Technology, Wuhan, 430000 China

**Keywords:** Medical insurance, Urban employee basic medical insurance, Social security card, Floating population, Settlement intention, Public health

## Abstract

**Background:**

Medical insurance and social security cards are an important incentive for the floating population to live a stable life in their current residence, but there has been little studies on their effect on settlement intentions. Therefore, the purpose of this paper was to study the impact of basic medical insurance for urban employees and application for personal social security cards on the settlement intentions of the floating population. With the increase of the desire to settle, the health management and the development of public health will be improved.

**Methods:**

Based on the 2017 survey data from the dynamic monitoring of China's floating population, we explored the influence of basic medical insurance for urban employees and social security cards on the floating population's settlement intentions. Additionally, this study also examined the comprehensive causal relationship, with social integration as the mediator variable. We used SPSS 21.0 software. The input method was used to analyze the influence of the above variables by binary logistic regression. Then we used AMOS22.0 software to establish the structural equation model of the relationship between the above three independent variables. Finally, we used bootstrapping method to analyze the direct effect, indirect effect and total effect of independent variables on settlement intention.

**Results:**

The settlement intention of members of the floating population after participating in basic medical insurance for urban employees was 23.2% higher than that of those who did not participate. The decision as to whether to apply for a personal social security card is related to their settlement intention. The standardized regression coefficients among social insurance and security, social integration, and settlement intention were positive values, and the Z values of the overall effect, indirect effect, and direct effect were all greater than 1.96; the confidence interval of the indirect effect did not include 0. We found that this model is a partial intermediary model, with an intermediary ratio of 10.66%.

**Conclusions:**

This article highlights the important impact of basic medical insurance for urban employees and individual social security cards on the floating population. The conclusions of this study provide suggestions for the government to use when designing policies to enhance the settlement intentions of the floating population and to improve the development of public health undertakings.

## Introduction

With the acceleration of economic development and urbanization, a large floating population has emerged in China. According to the < China Statistic Almanac > (2019), the floating population was 244 million in 2017 and 241 million in 2018 [1]. The "Statistical Bulletin of National Economic and Social Development 2019" released by the National Bureau of Statistics declared that there was a floating population of 236 million in 2019 [2]. Based on the current situation, the floating population in the future will maintain this considerable size [3]. The floating population is defined as individuals whose registered permanent residence is their original residence, and they live and work in a current residence that is not their registered permanent residence [4–6]. Settlement intention is defined as the thoughts of the floating population about their future relocation arrangements after they have been in their current residence for some time. Medical insurance is a social insurance system established to compensate workers for economic losses caused by disease risks, which comprises three schemes: Basic Medical Insurance for Urban Employees (BMIUE), the Rural New Cooperative Medical Scheme (RNCMS) and Basic Medical Insurance for Urban Residents (BMIUR) [7–9]. BMIUE was the medical insurance introduced in China in 1988. It is jointly paid by the unit and the employee; RNCMS was a form of community-based health insurance, was established and offered cover to rural residents in 2003. Its premiums are mainly subsidized by the local government; BMIUR, a local government-subsidized medical insurance scheme for the unemployed in urban areas, was launched on a pilot basis in 2007. In 2016, RNCMS and BMIUR were merged into medical insurance for urban and rural residents [10]. In this article, medical insurance refers to BMIUE. Social security cards are electronic certificates that provide labor security for workers who work in the fields. All people who participate in social insurance can apply for a social security card. The main items of social insurance include endowment insurance, medical insurance, unemployment insurance, work-related injury insurance and maternity insurance.

Researchers have shown that the individual characteristics of the floating population and the characteristics of the place of origin and current residence can influence the floating population's settlement intention, such as whether to purchase urban housing and housing conditions [11, 12], family migration [13], environment and regional differences [14, 15], education level, work status, and social integration [5]. However, little attention has been paid to the impact of medical insurance and social security cards on the floating population's settlement intentions.

The research on the medical security of the floating population is of great significance and value to the field of public health. If the floating population is not sure whether to participate in medical insurance at their current place of residence, this will pose a major challenge to the prevention and treatment of infectious diseases in the field of public health at their current residence [16, 17]. There is also research evidence that providing medical insurance to the floating population in their current residence will significantly improve the stability of the floating population's life and work [18, 19]. Ultimately, this will improve access to health services and the subjective well-being of the floating population [20]. In terms of BMIUE, BMIUE is jointly paid by units and individuals, which can reduce the economic burden of the floating population and help them settle down in the place where they live.

Social security cards can be used to verify the identity of the patient when they purchase medicine or medical treatment, to store personal account funds and to record the medical treatment of the insured, which will encourage migrants to settle where they live [21]. It can also handle job-seeking registration and unemployment registration procedures; Claiming unemployment insurance benefits; Applying for employment training; Apply for labor ability appraisal and apply for and enjoy treatment of industrial injury insurance; Deal with related labor and social security affairs on the net. It is clear that these elements of social security and medical services are closely related to the social integration of migrants in their places of residence [20, 22, 23].

Social integration refers to the process of integrating into a new environment, which is a multidimensional concept [24–27]. It is a hot spot in the fields of public health and social science, and it not only promotes the resettlement intentions of the floating population [28, 29] but also has health benefits [30–32].

Research on the willingness of the floating population to settle down will not only promote the development of urbanization [33] but also contribute to the control of infectious diseases and chronic noncommunicable diseases in their place of residence [34]. Finally, it will improve the level of public health services and accelerate social and economic development.

Therefore, the objective of this article is to use the 2017 survey data about the dynamic monitoring of China's floating population (Volume A) to analyze the influence of BMIUE and social security cards on the floating population's settlement intentions. To examine the comprehensive causal relationships, "social integration" was introduced as a mediator variable.

## Methods

### Data sources and Variables

The data in this paper are from the existing questionnaire survey in China floating population data platform [35]. The questionnaires were collected from a total of 169,989 floating population from 31 provinces (autonomous regions and municipalities directly under the Central Government) and Xinjiang Production and Construction Corps. All of them came from the inflow areas where China's floating population is relatively concentrated [36]. After deleting part of the missing data and replacing the mean value, 154,586 people were finally included in the analysis. The proportion is 90.9%.

The core independent variables of this article were BMIUE and social security cards, including the decision whether to participate in BMIUE and whether to apply for a personal social security card. The dependent variable was the settlement intention. This was measured according to whether the individual was willing to move their household registration to their current residence. The mediator variable was social integration. Social integration includes social, economic, cultural and other aspects of integration [37]. It is measured in terms of whether migrants agree to become part of the local population.

The control variables were as follows: (a) demographic characteristics (i.e., age, gender, marital status, the household registration system, and education level), (b) economic characteristics (i.e., average monthly total local expenditure over the past year and whether a labor contract has been signed), (c) flowing characteristics (i.e., flowing range and flowing time), (d) health education (i.e., whether to receive health education on occupational disease prevention, whether to receive health education on STD and AIDS prevention, and whether to receive health education on the prevention and treatment of chronic diseases). Studies have proven that the factors influencing the settlement intentions inflow factors, outflow factors, barriers between the inflow and outflow areas, and the floating population’s self-factors [37]. Therefore, these variables were also selected in this study.

### Statistical methods

#### Descriptive statistics and logistic regression analysis

Firstly, we used SPSS 21.0 software to describe the demographic indicators of the floating population, the participation in BMIUE, the application for social security card and the residence intention of the floating population by frequency and percentage. Then the input method was used to analyze the influence of BMIUE, social security card and social integration on settlement intention by binary logistic regression.

### Structural Equation Model (SEM)

We used AMOS22.0 software to analyze the influence path and effect of various factors on settlement intention. Then the goodness of fit of the model was evaluated by Index of Good Fit (GFI), Adjust the Index of Good Fit (AGFI), Comparative Fit Index (CFI), Root Mean Square Error of Approximation (RMSEA). The criteria for model fitting are GFI > 0.9, AGFI > 0.9, CFI > 0.9, RMSEA < 0.05 [38–40]. Remember, if the model fitting result is poor, the model needs to be improved in combination with the professional. Finally, we used bootstrapping method [41–43] to analyze the direct effect, indirect effect and total effect of independent variables on settlement intention. This method provided direction for interventions [44, 45].

## Results

### Descriptive analysis

According to Table 1, 39.9% of the floating population expressed their willingness to move their household registration system to the local area and settle there. This result is similar to the findings of previous research [46]. Of the respondents, approximately 77.7% did not participate in BMIUE, and 50.5% of the floating population had applied for a personal social security card. In addition, the survey found that the floating population had good social integration at their current residence (93.7%).

**Table 1:** Description of the variables included in the analysis (N = 154,586)

Variables	Categories	Frequency	Percentage (%)
Demographic characteristics			
Age	18–35 = 0	63,089	40.80
	35–60 = 1	84,032	54.40
	≥ 60 = 2	7465	4.80
Gender	Male = 0	79,577	51.50
	Female = 1	75,009	48.50
Marital status	Unmarried = 0	21,063	13.60
	Married = 1	128,038	82.80
	Divorce and others = 2	5485	3.50
The household registration system	Agriculture = 0	119,675	77.40
	Non-agricultural = 1	22,858	14.80
	Other = 2	12,053	7.80
Education	Elementary school and below = 0	26,358	17.10
	Junior high school = 1	67,181	43.50
	High school, technical secondary school = 2	33,785	21.90
	College and above = 3	27,262	17.60
Economic characteristics			
Average monthly total local expenditure (yuan)	< 1000 = 0	10,645	6.90
	1000–3000 = 1	76,673	49.60
	3000–5000 = 2	44,498	28.80
	> 5000 = 3	22,770	14.70
Labor contracts	Sign a contract = 0	89,323	57.80
	No contract signed = 1	65,263	42.20
Flowing characteristics			
Flowing range	Interprovincial = 1	74,875	48.40
	Intercity = 2	51,682	33.40
	Cross County = 3	28,029	18.10
Flowing time (years)	< 8 = 0	81,793	52.90
	8–14 = 1	43,688	28.30
	14–20 = 2	18,356	11.90
	> 20 = 3	10,749	7.00
Health education			
Health education in occupational disease prevention	No = 0	103,008	66.60
	Yes = 1	51,578	33.40
Health education on STD and AIDS prevention	No = 0	93,286	60.30
	Yes = 1	61,300	39.70
Health education on prevention and treatment of chronic diseases	No = 0	96,713	62.60
	Yes = 1	57,873	37.40
Insurance and Social security card			
Urban employee basic medical insurance	No = 0	120,176	77.70
	Yes = 1	34,410	22.30
Apply for a personal social security card	Did not apply = 0	76,535	49.50
	Apply = 1	78,051	50.50
Do you agree that I think locals are willing to accept me as a member?	Disagree = 0	10,380	6.70
	Agree = 1	144,206	93.70
Do you agree with move your household registration to the current residence?	Disagree = 0	92,931	60.10
	Agree = 1	61,655	39.90

Regarding demographic characteristics, the majority of the respondents were rural residents, married, and young or middle-aged men (95.2%), and nearly 43.5% of the respondents stopped their education in junior high school. In addition, 51.5% of the floating population was within the province of their registered residence, and their migration time was less than 8 years. Nearly 58% of them had signed labor contracts, and their average monthly expenditure in the past year on health care was 1000–3000 yuan. In terms of health education, the floating population received the most health education on STD and AIDS prevention, but their overall acceptance of health education was poor.

### Binary logistic regression results of BMIUE, social security card, social integration, and settlement intention

Table 2 shows the results of binary logistic regression, which shows that participation in basic medical insurance for urban employees (BMIUE) has an impact on the resettlement intention of floating population. Specifically, the settlement intention of individuals in the floating population participating in BMIUE was 23.2% higher than that of those who did not participate. During the single factor analysis, it found that applying for a personal social security card was related to the settlement intention. Moreover, the proportion of floating population agreeing to settle that they were already part of the society was 2.026-fold greater than that of the floating population that did not agree to settle. This demonstrated that social integration has a positive impact on the settlement intentions of the floating population.

**Table 2:** Logistic analysis of influencing factors of settlement intention

	B	S.E	Wals	df	Sig	Exp (B)	EXP(B) 95% CI	
							Lower limit	Upper limit
Age	− .016	.018	.776	1	.378	.985	.951	1.019
Gender	.105	.016	43.189	1	.000	1.110	1.076	1.145
Marital status	.088	.019	21.178	1	.000	1.092	1.052	1.134
Education	.250	.010	642.384	1	.000	1.285	1.260	1.310
The household registration system	.249	.013	362.815	1	.000	1.283	1.251	1.316
Average monthly total local expenditure	.174	.010	278.571	1	.000	1.190	1.166	1.215
Labor contracts	− .056	.019	8.408	1	.004	.945	.910	.982
Flowing range	− .304	.011	772.995	1	.000	.738	.723	.754
Flowing time	.167	.010	306.207	1	.000	1.182	1.160	1.204
Basic medical insurance for urban employees (BMIUE)	.209	.021	97.274	1	.000	1.232	1.182	1.284
Apply for a personal social security card	.020	.019	1.090	1	.297	1.020	.983	1.059
Health education in occupational disease prevention Health education	− .151	.022	47.978	1	.000	.859	.823	.897
on prevention and treatment of chronic diseases	.121	.023	29.038	1	.000	1.129	1.080	1.180
Health education on STD and AIDS prevention	.016	.023	.530	1	.467	1.017	.973	1.062
Do you agree that I think locals are willing to accept me as a member?	.706	.033	456.131	1	.000	2.026	1.899	2.161
Constant	− 1.823	.053	1168.340	1	.000	.162		

### Structural Equation Model and the mediating effect of social integration

**Fig. 1:**
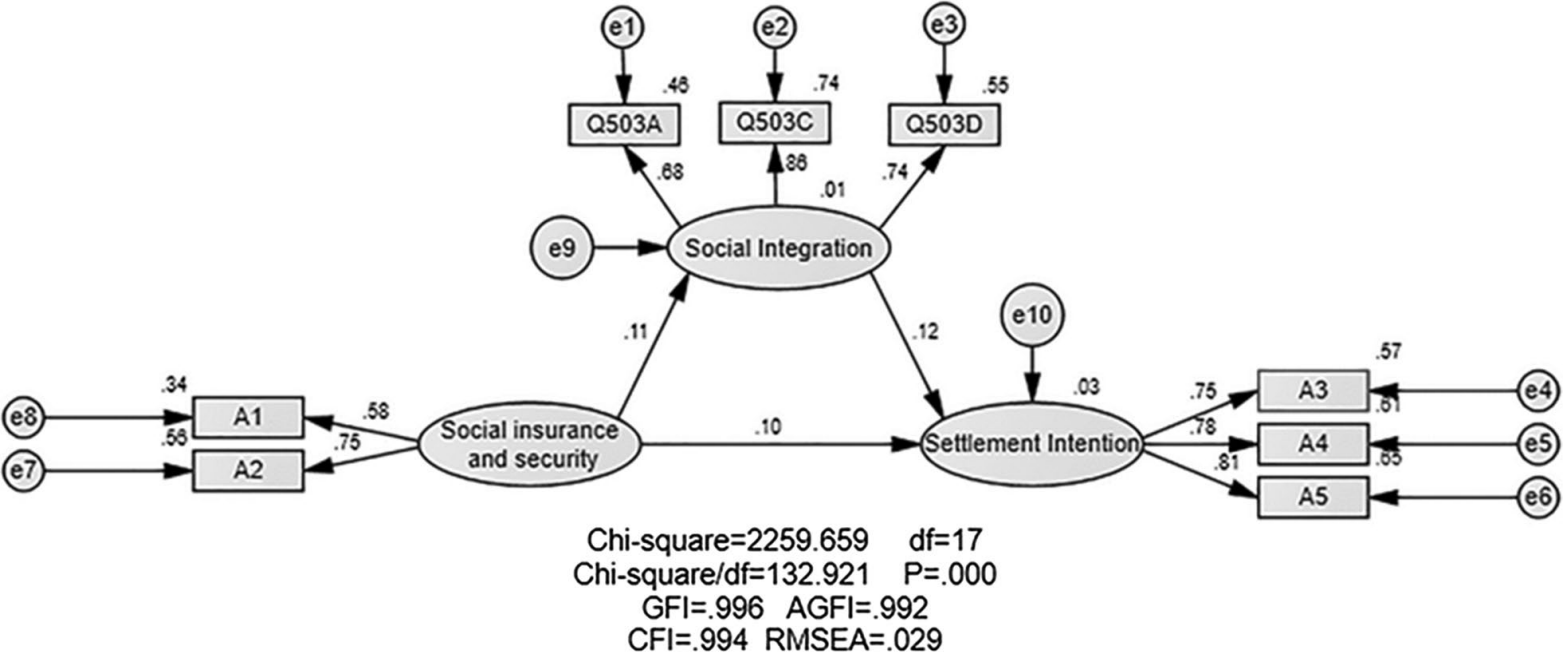
Structural equation model Note. Rectangles and ovals represent observed and latent variables, respectively. e represents residual variance. AGFI: Adjust the Index of Good Fit. CFI: Comparative Fit Index. RMSEA: Root Mean Square Error of Approximation. p values < 0.01: significant correlation between variables. A1: BMIUE; A2: Apply for personal medical insurance cards; A3: Health education in occupational disease prevention; A4: Health education on prevention and treatment of chronic diseases; A5: Health education on STD and AIDS prevention; Q503A: Do you agree with the statement I like the city/place where I live now? Q503C: Do you agree with the statement that I would like to be a part of the local people? Q503D: Do you agree with the statement "I think the local people are willing to accept me as a member"?

The structural equation model is shown in Fig. 1. SEM is composed of three latent variables: social insurance and security, social integration and settlement intention. Figure 1 shows that the standardized regression coefficients among social insurance and security, social integration, and settlement intention are positive values, which means social insurance and security and social integration have a positive impact on the resettlement intention of floating population. According to the model results, the factor loading coefficient of each observed variable is greater than 0.5. And the values in Table 3 are positive, which indicates that each variable is statistically significant.

**Table 3:** Regression weights

			Estimate	S.E	C.R	*P*	Label
Social integration	<---	Social Security	.115	.004	26.216	***	
Settlement intention	<---	Social Security	.099	.004	25.070	***	
Settlement intention	<---	Social integration	.109	.003	38.361	***	
A3	<---	Settlement intention	1.000				
A4	<---	Settlement intention	1.063	.004	268.101	***	
A5	<---	Settlement intention	1.111	.004	269.905	***	
A2	<---	Social Security	1.000				
A1	<---	Social Security	.642	.019	33.247	***	
Q503A	<---	Social integration	1.000				
Q503C	<---	Social integration	1.387	.006	239.244	***	
Q503D	<---	Social integration	1.181	.005	241.402	***	

A1: BMIUE; A2: Apply for personal medical insurance cards; A3: Health education in occupational disease prevention; A4: Health education on prevention and treatment of chronic diseases; A5: Health education on STD and AIDS prevention; Q503A: Do you agree with the statement I like the city/place where I live now? Q503C: Do you agree with the statement that I would like to be a part of the local people? Q503D: Do you agree with the statement "I think the local people are willing to accept me as a member"? ***p < 0.01

Table 4 shows the goodness-of-fit indices. The fitness indexes (GFI/AGFI/CFI) were all greater than 0.9, and the RMSEA was less than 0.05. This shows that the model has a good fitting effect.

**Table 4:** Evaluation Index of Model Fit

Absolute fit index	Fit standard	Fitness index
NC(χ2/*df*)	1 < NC < 3	132.92
*P*	The smaller the better	0.00
GFI	> 0.9 Good fit	1.00
AGFI	> 0.9 Good fit	0.99
CFI	> 0.9 Good fit	0.99
RMSEA	< 0.05 Good fit	0.03

GFI: goodness-of-fit index. AGFI: adjusted goodness-of-fit index. CFI: comparative fitness index. RMSEA: root mean square error of approximation

Mediation analysis is an important tool for statisticians to study causality. The intention is to study whether or to what extent the independent variable acts on the dependent variable via the mediator variable and to clarify the direct effect, indirect effect, and total effect [41, 42]. To further clarify the causal relationship, the study analyzed the intermediary effect by using the bootstrapping method. The reliability of the direct effect, indirect effect, and total effect was 0.600, 0.804, and 0.823, and the validity was 0.500, 0.695, and 0.719, respectively. The results of the mediation effect using the bootstrapping method are presented in Table 5. It was found that the Z values of the overall effect, indirect effect, and direct effect were all greater than 1.96, and the confidence interval of the indirect effect does not include 0, indicating that both direct and indirect effects exist in this model. This model is a partial intermediary, with an intermediary ratio of 10.66%.

**Table 5:** Direct and indirect effects of social insurance and security cards on settlement intention

Variables	Estimate	S.E	Z	Bootstrapping			
				Bias-corrected 95% CI		Percentile 95% CI	
				Lower	Upper	Lower	Upper
Total effect							
Insurance and security-Settlement intention	0.122	0.004	30.500	0.105	0.118	0.105	0.118
Indirect effect							
Insurance and security-Settlement intention	0.013	0.001	13.000	0.011	0.014	0.011	0.014
Direct effect							
Insurance and security-Settlement intention	0.109	0.003	36.333	0.104	0.115	0.104	0.115

## Discussion

After binary logistic regression analysis and structural equation model mediation effect analysis, it was concluded that BMIUE, personal social security card application and social integration not only directly promote the settlement intentions of the floating population but also promote the settlement of the floating population in their current residence through the intermediary variable of social integration.

### BMIUE, social security cards, and settlement intentions

Participating in BMIUE in the current residence is very important for the future settlement intentions of the floating population. Participation in BMIUE has a positive impact on the settlement intentions of the floating population, and the impact on the settlement intention is greater than that of participating in other types of insurance [47], which is consistent with the findings of previous studies [48, 49]. In addition, researchers previously found that the participation of the floating population in BMIUE in big cities would contribute to their intentions to settle down [50]. More importantly, participating in BMIUE can improve the risk resistance of the floating population at their current residence, reduce their cost of living and reduce their economic burden to a certain extent [51–53]. BMIUE is paid for by both the employer and the employee [54], and the employer pays more than each individual. Employees only need to pay for a certain number of years to enjoy higher rates of medical insurance reimbursement for life. Therefore, this will enhance the floating population's intention to settle down to some extent. Even without considering the preferences of migrant workers, the participation in medical insurance still has a positive impact on the willingness of the floating population to settle down [55–57].

In 2009, the state promulgated the Interim Measures on the Continuity of the Basic Medical Security for Floating Employed Persons, which clearly stipulates that local governments should not set up barriers to the participation of the floating population for reasons such as having registered permanent residence. If rural household registration personnel are employed in urban units and have stable labor relations, the employing units shall go through registration procedures in accordance with the provisions of the Interim Measures for the Administration of Social Insurance Registration and participate in the basic medical insurance for urban workers in the place of employment. Other mobile employment, can voluntarily choose to participate in the new rural cooperative medical care in the place of household registration or the urban basic medical insurance in the place of employment, and according to the relevant provisions to the new rural cooperative medical care agency in the place of household registration or the social (medical) insurance agency in the place of employment for registration procedures; Urban basic medical insurance ginseng protect personnel to cross flow as a whole the area of employment, without receiving unit, the individual should be suspended within 3 months after the original relationship of insurance of primary medical treatment to the new employment to social (medical) insurance agency organization is dealt with register formalities, participate in BMIUE in accordance with local regulations or BMIUR [58]. However, according to the survey results of this paper, there are still some people who do not participate in BMIUE.

The reason that produces this kind of result may be floating population attends insurance in place of origin, the insurance after transforming the job is interrupted, cannot timely pay is sure; The floating population is not stable in their current residence, even in the informal sector of employment; Reimbursement procedures are complicated and the reimbursement ratio is lower than that of the place of origin. In violation of the Labor Contract Law, some units do not sign labor contracts with the floating population and refuse to pay insurance premiums [59]; The provinces where the floating population flows into have weak economic strength and insufficient medical insurance funds [60].

Social security cards had a positive effect on the settlement intentions of the floating population, which is consistent with previous studies [61, 62]. It has been documented that the issuance of and application for a social security card is an important push to relieve the worries of the floating population and promote the construction of new urban areas. The social security card is an important guarantee for the floating population that encourages them to integrate into their current residence and live a stable life [63]. Migrants can receive a social security card as long as they are insured in their place of residence. These cards are used for the identification of their medical insurance, and they are also used to record the basic information of the insured persons, payment status, treatments, payment of medical funds and other information [64]. In addition, it can also handle job-seeking registration and unemployment registration procedures;

Claiming unemployment insurance benefits; Applying for employment training; Apply for labor ability appraisal and apply for and enjoy treatment of industrial injury insurance; Deal with related labor and social security affairs on the net. Therefore, even though it had an effect on the settlement intention in the single-factor analysis and no effect on the regression analysis, this study still included the social security card in the final model. But we have to note that half of them still don't have a social security card. The reasons may be: The time limit of insurance payment is less than 3 months [65]; Change your name, photo and service bank before you can apply for again [66]; Failing to renew your social security card before it expires [67].

### Social integration and settlement intention

Social integration has become an important factor affecting the settlement intentions of the floating population [68]. With the development of urbanization, the floating population has paid increasing attention to their acceptance in the local society [50]. The "people-centered" urbanization policy put forward by the Chinese government in 2014 shows that urbanization is the urbanization of people, and the degree of social integration is closely related to the degree of the acceptance of the floating population by the local people [69–71]. This is consistent with the selection of the social integration measurement criteria in this paper because the current residence provides abundant social resources and conditions for the floating population, which will help them integrate into the local life as soon as possible [72]. The longer the influx takes, the stronger the positive effect of social integration on settlement intentions [73].

### The mediating effect of social integration

Participation in BMIUE in the place of residence is an important indicator to measure social integration [74]. Participation in BMIUE will promote the social integration of the floating population in the local area [75], which is consistent with the results of this study. This may be because the eligibility for BMIUE indicates that the floating population has a fixed source of income and a stable work unit in their current place of residence, and their living conditions are relatively good, which can meet the basic living needs of the floating population. Participating in social insurance is the only requirement for obtaining a social security card. Therefore, BMIUE and social security cards, which are closely related to social security, are important factors affecting the social integration of the floating population. They will increase the sense of belonging of the floating population in their current place of residence and ultimately improve their willingness to settle down [76].

### Settlement intention and public health

An increase in permanent residence will not only improve the health management level of the floating population in their place of residence but also promote the improvement of public health. The residents' health records are an important part of the twelve contents of the national basic public health service, which takes personal health as the core and satisfies the residents' self-care and health management [77]. Studies have shown that long-term settlement intention can significantly promote the service utilization of the health records of the floating population [78], which is beneficial to the physical health of the floating population. At the same time, settlement intention will also improve the level of public services [79–81]. As their willingness to stay increases, so does the prevention of communicable and noncommunicable diseases in the place of residence. This is because the willingness to stay promotes urbanization, which leads to an increase in global risk factors for infectious and noncommunicable diseases [82–84]. Specifically, China's public health service system for the floating population is not sound, and there is a lack of disease data from the floating population on infectious diseases and chronic noncommunicable diseases, making it difficult to establish a disease surveillance system [84–86]. This leads to the absence of prevention and the control of disease in vulnerable groups such as the floating population in China, and makes infectious diseases such as tuberculosis an important threat to public health [84, 85]. For example, in the COVID-19 epidemic in 2019, the floating population will be at increased risk of COVID-19 infection due to their high rates of chronic disease comorbidities [87–89]. Additionally, because of their different medical insurance coverage, some vulnerable groups will not be able to timely treatment [84, 88]. As a result, some countries, including China, have taken measures to control migration flows to control the COVID-19 epidemic [23].

To sum up, BMIUE and social security cards, as part of social security, have a direct positive impact on the floating population. Social integration, which is closely related to social security and public services, also has a direct positive impact on the resettlement intentions of the floating population. This study proved the mediating effect of social integration through constructing a model; BMIUE and social security cards can promote the settlement intentions of the floating population through social integration. The increase in permanent residence will eventually improve the health management level of the floating population and promote the development of public health.

However, this study also has some limitations. The results of this study are based on mining of existing data. Due to the limited variables in the original data, the reliability of some indicators and the intermediary ratio is low. If one can add new effective variables in the future, the explanation of the influence of medical insurance and social security cards on the settlement intentions of the floating population will be more complete.

## Conclusions

In this paper, the dynamic monitoring data of China's floating population in 2017 were used to study the influence of BMIUE and social security cards on the settlement intentions of the floating population, taking social integration as a mediating variable. According to the mediating effect method of the structural equation model, BMIUE and social security cards not only have a direct positive impact on the floating population but also indirectly affect their settlement intentions through social integration. Therefore, the government should further improve the insurance policies of the floating population in their current place of residence, encourage the floating population to integrate into society, and further enhance their intention to settle down. At the same time, settlement intention will also promote the development of public health and economic development. Although the data used in this paper were obtained from developing countries, this result also provides a theoretical basis for improving population migration policies internationally.

## Data Availability

The datasets generated and analysed during the current study are available in the Migrant Population Service Center, National Health Commission P.R. China repository, http://www.chinaldrk.org.cn/wjw/#/data/classify/population/yearList.

## Abbreviations

BMIUE: Basic medical insurance for urban employees
BMIUR: Basic Medical Insurance for Urban Residents
RNCMS: Rural New Cooperative Medical Scheme
GFI: Index of Good Fit
AGFI: Adjust the Index of Good Fit
CFI: Comparative Fit Index
RMSEA: Root Mean Square Error of Approximation
SEM: Structural Equation Model
